# Further experiments and remarks regarding the possible formation of blood stains on the *Turin Shroud*: stains attributed to the nailing of the hands

**DOI:** 10.1007/s00414-024-03166-7

**Published:** 2024-02-10

**Authors:** Anna Heger, Lisa König, Alexandra Reckert, Melanie Schröder, Ferhat Kiliç, Farahs Emami, Stefanie Ritz-Timme

**Affiliations:** Institute of Legal Medicine, University Hospital Düsseldorf, 40225 Düsseldorf, Germany

**Keywords:** Turin Shroud, Nailing wound, Scourge wounds, Blood stain pattern

## Abstract

The formation of red discolorations (‘blood stains’) on the *Turin Shroud* (TS), a Christian relic believed to be the burial cloth of Jesus of Nazareth, is controversially discussed. We performed experiments to identify possible explanations for the formation of the stains on the hands and forearms of the *Turin Shroud Man* (TSM). In preliminary non-standardised experiments, after applying blood to the dorsal and palmar side of the probands’ wrists, they moved their arms around at their own discretion to provoke blood flows as similar as possible to those on the TS. A blood stain pattern similar to that on the left wrist could be provoked by slowly turning the wrist to the ulnar side. In contrast, a branched pattern of multiple streaks, as depicted on the forearms, was difficult to reproduce. In a standardised test setup, the probands moved their dry, dirtied, or oiled arms jerkily in a predetermined sequence of movements. More body hair only slightly facilitated the formation of a branched pattern. On oiled skin, however, the formation of branches was significantly facilitated. This may support the hypothesis that the blood stains on the forearms were formed by moving the body between the unnailing and the burial. The formation of a branched pattern seems feasible if the arms were moved jerkily and were possibly exposed to water and oils postmortem (e.g. transporting the washed and oiled body). Nevertheless, the well-defined blood stains with multiple branchings are difficult to explain. Additionally, the blood stains on the forearms may have originated from deep scourging wounds, where dried blood was again mobilised by water (and oil). We are aware that no reliable conclusions about the formation of the ‘blood stains’ on the TS can be drawn from our findings. However, they may contribute to the discussion on this topic.

## Introduction

The *Turin Shroud* (TS), a Christian relic believed to be the burial cloth of Jesus of Nazareth, exhibits red discolorations that have been interpreted as blood stains derived from injuries due to torment and crucifixion.

Even though these red discolorations have not yet been analysed by modern scientific methods, for the purpose of this paper, we will not challenge the interpretation as blood stains. Our research focuses exclusively on the question, how the formation of such blood stains could theoretically be explained.

Numerous hypotheses regarding the formation of the blood stains have already been published (for an overview, see [1]) and have, in part, been heavily discussed between ‘TS believers’ and ‘TS sceptics’ (e.g. [2]). Since we felt that some of the published hypotheses should be investigated further, we conducted experiments that may contribute to the discussion of the origin of the blood stains on the TS.

In our previous work [1], we identified theoretically possible explanations for the formation of blood stains similar to those that have been attributed to injuries caused by the *crown of thorns*, the *belt of blood* and the serum ring in the area of the *lance wound* of the *Turin Shroud Man* (TSM). Continuing this work, we now investigated the blood stains attributed to the *nailing of the hands* to the cross.

**Fig. 1:**
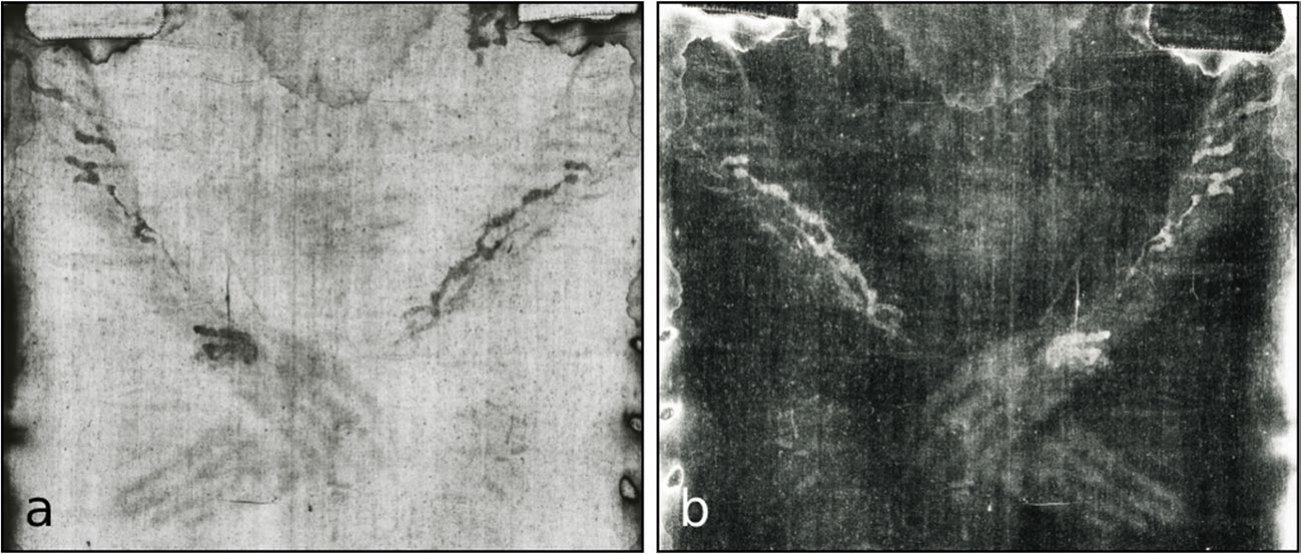
‘Blood stains’ on the wrists and forearms of the *Turin Shroud Man* (TSM). **a** Positive image, **b** negative image (photo from http://www.shroudphotos.com, ©Vernon Miller, 1978; cropped by the authors)

The Gospels (at least indirectly) state that Jesus’ hands were nailed to the cross (John 20:25 [3]). Bordes et al. [4] concluded from their experiments with cadavers that the most likely region for the nail insertion was ‘through the carpal bones of the wrist’. The blood stains on the left wrist and the forearms on the TS (see Fig. 1) have been related to these *nailing wounds*.

Svensson and Heimburger [5] described these blood stains “as in an autopsy room”: “Two large blood flows emanate from a single puncture wound in the left wrist. Apart from a brake, they seem to wriggle along the forearm to the elbow. The flow changes all the way in two or three distinct directions. The same phenomenon is seen on the right arm …”. A more detailed description was given by Bevilacqua et al. [6] after the analysis of high-resolution images of the TS. On the left wrist, they described “two trickles” that “are directed towards the ulnar side spreading apart at an angle of 38° +/- 2°” with reference to a line drawn between the 3^rd^ and 4^th^ metacarpal. The blood stains on the left forearm are described as trickles of blood with “a diagonal course from the wrist to the medial part of the elbow, with an angle of 38° +/- 2° (like that on the wrist) with small streams slanting towards the outside of the forearm, due to the radial flexion of the hand”. The blood stains on the right forearm are reported as “almost unidirectional, coaxial to the arm”.

Many authors have interpreted the blood stains on the left wrist and the forearms as a result of a vital bleeding at the cross from the *nailing wounds* and have deduced the position of the arms on the cross from the characteristics of these stains. According to Svensson and Heimburger [5], “the specific directions of blood flows from wrists and forearms, following gravity, make sense, if the arms have been stretched upward at an angle of about 65° and from this position have slightly changed the positions in a frontal plane…”. The assumption of an arm position of approximately 65° at the time of the blood flow is shared by other authors [7, 8]. Bucklin [8] explains the “angulation of the stain on the wrist” (angle about 10°) and the “divergence of the streams” at the forearms by two positions “maintained by the victim during the period of blood flow”, assuming an elevation of “his body by directing his weight toward the feet and then changing position to permit the full body weight to be supported by the wrists”. A similar mechanism is assumed by Barbet [7], who describes an “alteration of sagging and lifting, of asphyxia and momentary relief” in the phase of agony. Lavoie et al. [9] pointed to some similar features of the blood stains seen on the forearms and the (fore)head, all of which being “dark and distinct” and obviously having formed “on the body of a man who died in the position of crucifixion”. Based on experiments regarding the feasibility of a blood transfer from blood clots on cling film or skin to linen, the authors conclude that these “dark and distinct” blood stains are the results of a transfer from moist clots due to an active bleeding “near the time of death”. The “intact and well-defined” shape of the blood stains is explained by an “extreme care… in the removal of the body from the vertical position to its subsequent horizontal placement in the Shroud”.

In contrast to the authors who support the hypothesis of a vital bleeding on the cross as origin of the blood stains, others discuss a postmortem formation. Zugibe [10] explains the “peculiar bifurcation pattern at the back of the hand” by “the formation of rivulets of blood running to the two sides of the ulnar styloid protuberance … after removing the nail from the wrist…”. Bevilacqua et al. [6] believe these stains have only formed after the wrists were unnailed from the cross and “simply due to the movements undergone by the arms” during the preparation of the burial. They explain the “significant drain of blood on the forearms” as caused by a possible injury of the ulnar artery, and the course of the blood stains at the forearms as a result of possibly raising “his arms to prevent the blood spilling from the holes on the ground”. Zugibe [10] as well as Bevilacqua et al. [6] assumed that the body was washed according to Jewish rules, whereby the postmortem blood (from the unnailing) on the left wrist and the forearms was not removed “because according to Jewish custom it was forbidden to touch post-mortem blood as it would make the person “*impure*” [6]. Faccini et al. [11] state that the “wrist blood stain pattern” could have been formed at different occasions between crucifixion and burial, on the cross or when the TSM was moved from the cross to the sepulchre.

Using a “BPA approach to the shroud of Turin”, Borrini and Garlaschelli [12] performed experiments by fixing a transfusion cannula to the wrist (Destot’s space) of a living volunteer and by trickling whole blood (with anticoagulant) to simulate the dripping from a puncture-type injury. According to their results, “the angle between the arm and the body must be greater than 80° and smaller than 100°, in order for the rivulets to flow from the wrist towards the elbow on the outer part of the forearm, as it appears on the Shroud” (with 0° = arm held horizontally, 90° = arm held vertically). In contrast, they report a required angle of approx. 45° for the formation of the short rivulets at the back of the left hand. The authors conclude that “the blood patterns on the forearms and on the back of the hand are not connected; they would have to occur at different times, and (should the Shroud be authentic) a particular sequence of events or movements would have to be imagined to account for these patterns”. This work of Borrini and Garlaschelli [12] has been heavily criticised by Fanti and Malfi [2], Sanchez Hermosilla et al. [13] and Rucker [14] for several reasons. Some of their main points were the usage of blood with an anticoagulant in the experiments and especially the lack of consideration and discussion of the effects of many possible positions of the TSM during crucifixion, unnailing, the taking down from the cross, the transport to the sepulchre, and the burial.

Faccini [15] pointed out that also “the presence of *scourge wounds* could have affected the shape of the blood flows from the wrist wounds”.

At the moment, there is no direct access to the Shroud, to physical samples of the Shroud and not even to all existing photographs of the Shroud. All information regarding the circumstances of Jesus Christ and the events after his death stem only from the Gospels that were written at least 70 years later [16]. Therefore, it is clearly not possible to exactly reconstruct the origin of the blood stain pattern on the TS. With that in mind, we performed some experiments very similar to our previous approach [1] to identify possible explanations for the formation of the blood stains on the hands and forearms of the TSM.

## Materials and methods

**Fig. 2:**
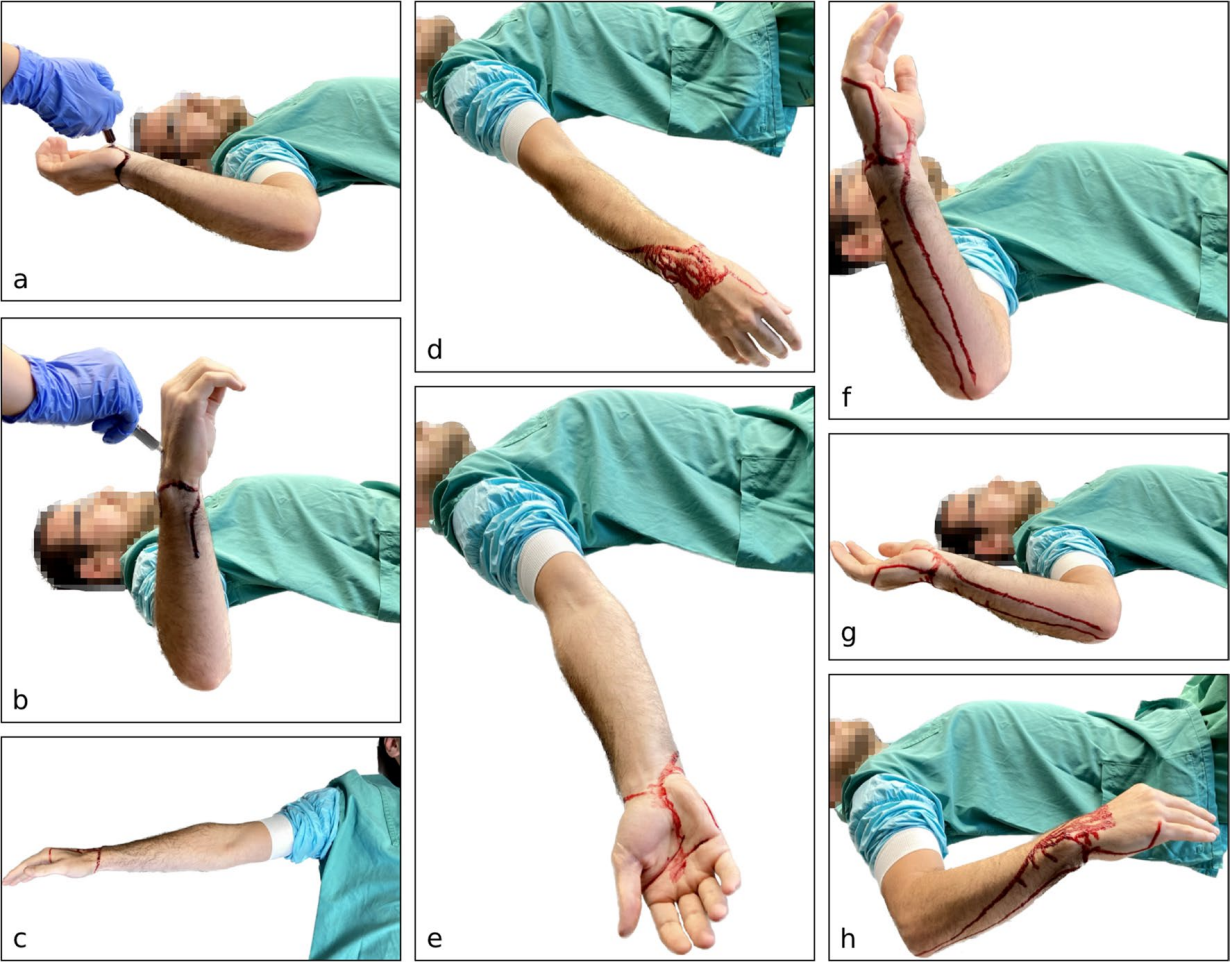
Experimental setup (for details, see experiment Ib; for test results, see Fig. 3). Proband in supine position on a table. **a**, **b** Application of blood from a syringe, **c**–**h** sequence of arm movements in the standardised experiments

For each experiment, blood samples were taken from live probands (one female proband with slender and hairless forearms, two male probands with muscular and hairy forearms). No anticoagulants were added. Two millilitres of either undiluted or diluted whole blood (dilutions 1:1 with saline solution or tap water) was applied dropwise to both the palmar and dorsal side of the wrist as depicted in Fig. 2a and b. The experiments were repeated at least twice.

I. **Experiments with focus on the branched blood stain pattern on the forearms**
  - In a preliminary set of non-standardised experiments, the probands were asked to move their arms freely at their own discretion after the application of blood to provoke blood flows as similar as possible to those on the TS.
  - In further experiments, the probands were asked to move their arms in a predetermined standardised sequence, as depicted in Fig. 2. The probands lay in supine position on a table. After each of the following movements, their arm was held in the resulting position for 10 s.
    - Arm abducted and rotated backward at the shoulder joint, bent 90° at the elbow, the wrist in neutral position. Application of blood (approx. 2 ml) from a syringe to the palmar side of the wrist.
    - Arm abducted and rotated forward at the shoulder joint, bent 90° at the elbow, the wrist in neutral position. Application of blood (approx. 2 ml) from a syringe to the dorsal side of the wrist.
    - Arm stretched out at the elbow in a swift and jerky motion.
    - Arm relaxed, hanging loosely down the side of the table.
    - Quickly and jerkily alternating between supination and pronation of the forearm (2×).
    - Arm moved slowly back to position (b).
    - Arm quickly and jerkily rotated backward at the shoulder joint.
    - Arm quickly and jerkily rotated forward at the shoulder joint.
    - Arm relaxed, hand put on/near groin, forearm in pronation (not depicted).
    With this standardised experimental setup, the impact of the following influence factors on the formation of blood flow patterns was examined:
    **Individual conditions (e.g. anatomical features, hair)**
      Identical experiments were performed with:
      a male proband with muscular and hairy forearms
      a female proband with slender and hairless forearms
    **Dilution of blood with water**
      Identical experiments were performed with:
      whole blood, undiluted, no anticoagulants
      watery dilutions of whole blood (1:1)
    **Water and dirt on the skin**
      Identical experiments were performed with:
      dry and clean forearms
      wet and dirtied forearms (sand, soil)
    **Oily substances on the skin**
      Identical experiments were performed with:
      dry and clean forearms
      forearms after applying a skin protection cream containing paraffin oil (Stokoderm® Aqua PURE)
II. **Experiments with focus on the blood stain on the left wrist**
  After applying approx. 2 ml of whole blood to the dry and clean wrist, the probands were asked to provoke a blood stain as similar as possible to that on the left wrist on the TS.

## Results

### Experiments with focus on the branched blood stain pattern on the forearms

Moving their arms freely in such a manner as to provoke blood flow patterns as close as possible to those on the TS (experiments Ia), the probands had great difficulty to recreate a similarly branched pattern and even more so a pattern of multiple (parallel) streaks of similar width on nearly the entire dorsal aspect of the forearm. In all those experiments, the most effective way to provoke bifurcations of blood flows were jerky movements of the arms, whereby the blood stream or a drop of blood collecting at the end of a stream, when the blood flow slowed down, was given the impulse to flow in another direction.

With these results in mind, we planned standardised experiments (Ib, see Fig. 2) to test the impact of different influence factors on the formation of blood flow patterns, leading to the following observations:

- **Influence of individual conditions (e.g. anatomical features, hair, blood)**

**Fig. 3:**
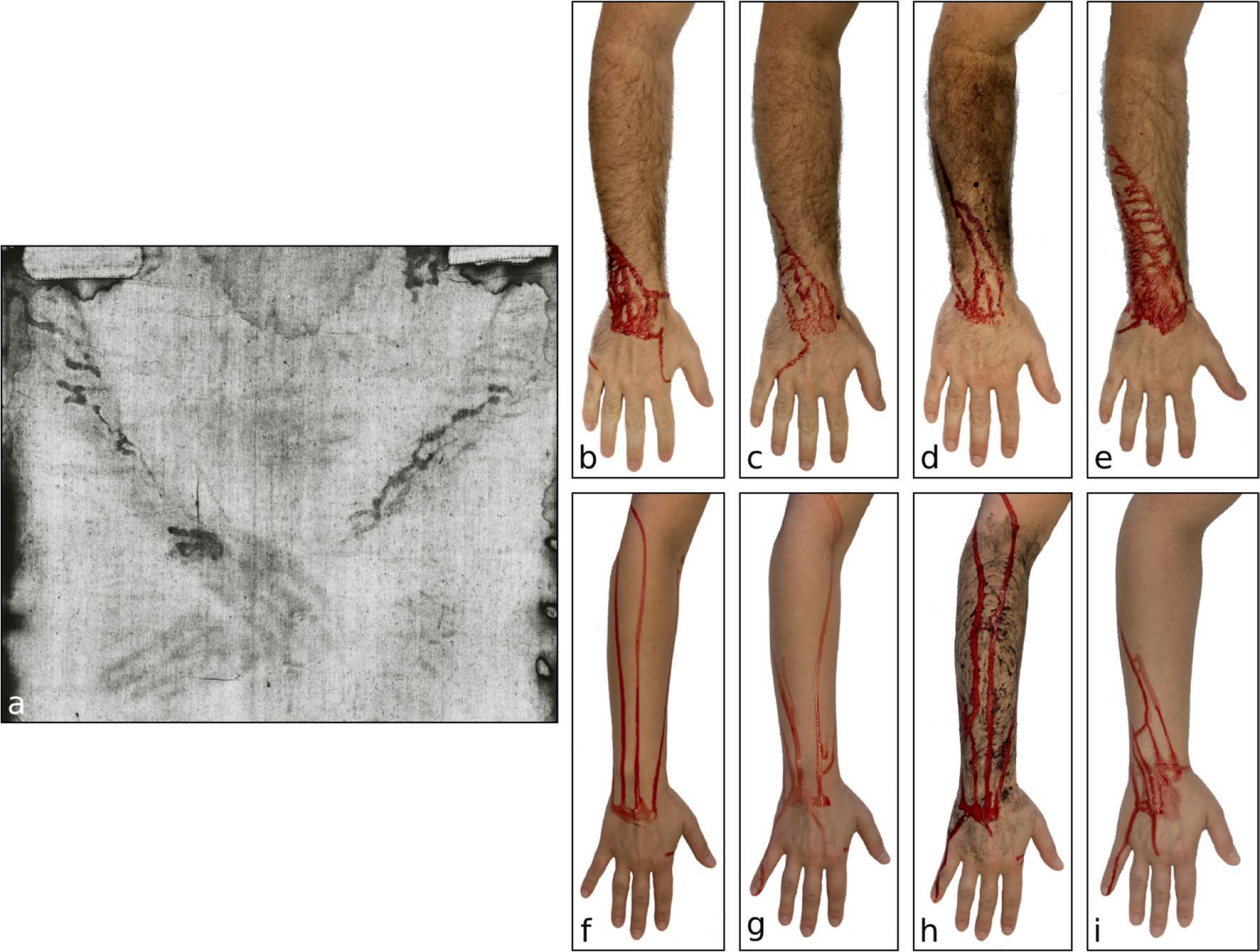
Patterns of blood rivulets on the probands’ forearms (**b**–**i**) after each standardised experiment (for details, see experiment Ib; for test setup, see Fig. 2), as compared to the pattern on the TS (**a**). **a** Corresponding ‘blood stains’ on the TS (photo from http://www.shroudphotos.com, ©Vernon Miller, 1978; cropped by the authors), **b** male forearm, dry, whole blood, **c** male forearm, dry, diluted blood (watery dilution, 1:1), **d** male forearm, wet and dirtied, whole blood, **e** male forearm, oiled, whole blood, **f** female forearm, dry, whole blood, **g** female forearm, dry, diluted blood (watery dilution, 1:1), **h** female forearm, wet and dirtied, whole blood, **i** female forearm, oiled, whole blood

The way blood flowed from the points of application towards the elbow proved to be quite differing between the probands (see Fig. 3b–e vs. f–i). Under ‘baseline’ conditions (whole blood to dry skin), the blood flowed much faster on the forearms of the female proband (with slender and hairless forearms) when compared to the male probands, and without any branchings or bifurcations, not even after jerkily moving the arms in different directions. The male probands, however, were able to provoke some branching by such jerky arm movements. In all probands, blood applied to the dorsal part of the wrist flowed rapidly to the ulnar side of the forearms. On the dorsal side, the bloody rivulets never reached the proximal forearms.

- **Influence of adding water to the blood**

The dilution of blood with water resulted in a faster flow, narrower rivulets and a slightly higher tendency for the formation of a branched flow pattern (see Fig. 3c and g).

- **Influence of applying water and dirt to the skin**

The blood flow pattern on wetted and dirtied forearms were more longitudinal and appeared to be even less branched, when compared to the findings on clean and dry skin (see Fig. 3d and h).

- **Influence of applying oily substances to the skin**

On oiled skin, the blood flowed much faster and had a high tendency to form multiple branchings; the rivulets also appeared much wider than on dry skin (see Fig. 3e and i).

### Experiments with focus on the larger blood stain on the left wrist

**Fig. 4:**
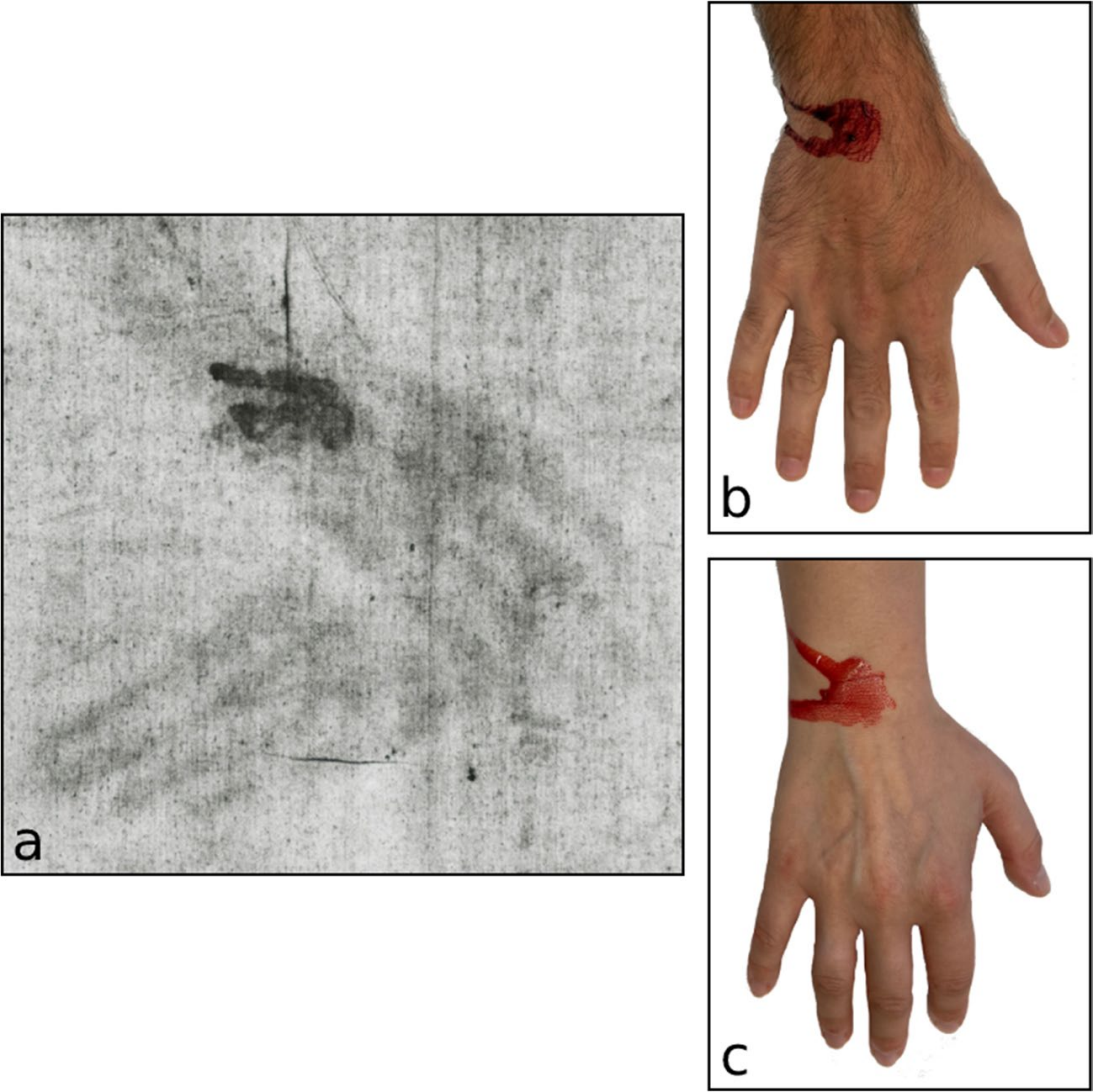
Patterns of blood pools and rivulets on the probands’ wrists (**b**, **c**), provoked by slow arm movements (for details, see experiment II), as compared to the pattern on the TS (**a**). **a** Corresponding ‘blood stains’ on the TS (photo from http://www.shroudphotos.com, ©Vernon Miller, 1978; cropped by the authors), **b** male wrist, dry, whole blood, **c** female wrist, dry, whole blood

A blood flow pattern or blood stain similar to that on the left wrist of the TSM could be provoked (after application of blood to the dorsal part of the wrist) by moving hand and forearm upwards to a slanted position, approx. 45° to the horizontal plane, and slowly turning it to the ulnar side of the wrist (see Fig. 4b, c); jerky movements of the arms were not necessary for the simulation of this pattern.

## Discussion

Blood stain pattern analysis is a special field of expertise in forensic science and forensic pathology. Even for experienced forensic experts, the blood stain pattern on the TSM’s hands and forearms appears to be unusual, especially regarding the multiple branches of almost uniform blood streaks spanning nearly the entire dorsal aspect of the forearm (see Fig. 1). These blood stains are remarkable, as they are very clearly demarcated with well-defined edges—similar to the marks on the head, as already emphasised by Lavoie et al. [9].

Our experiments were planned with the aim to find out whether the formation of such an unusual pattern of blood stains is in some way conceivable and further yet, which conditions hinder or facilitate this formation. Preliminary experiments were conducted to get a first idea of how such an unusually branched blood flow pattern may be provoked. We tackled this task by asking the probands to move their arms around freely at their own discretion—after blood had been applied to their wrists—to provoke blood flows as similar as possible to those on the TS. Obviously, these experiments could not be standardised, but they led to findings that more standardised experiments could be based on.

The subsequent standardised experiments (see Fig. 2) were aimed at the questions, if and how different conditions or influence factors (anatomical features, musculature, body hair, dirt, water, oily substances) had any impact on blood flow patterns on the forearms and, furthermore, if any of the different factors tested would facilitate the formation of a pattern similar or close to the one on the TS. Of course, many more potentially influencing factors are conceivable (e.g. individual coagulability). The selection of influence factors examined was based on the following considerations: In view of the account of events in the Gospels, one must assume that the body of the TSM must have been very dirty; dirt and/or soil (mixed with sweat) changes the surface texture of the skin and may therefore have affected the way the blood flows over the skin. Additionally, we considered the possibility of blood flow patterns being altered by a postmortem ritual washing (ablution) using water and “oils” (John 19:39+40: “And there came also Nicodemus, which at the first came to Jesus by night, and brought a mixture of myrrh and aloes, about an hundred pound weight. Then took they the body of Jesus, and wound it in linen clothes with the spices, as the manner of the Jews is to bury” [3]). Whether the TSM was indeed washed postmortem (possibly in an atypical way due to a short time left before the Shabbat began (Luke 25:54 [3])) has been controversially discussed [1, 10, 17]. The findings of Zugibe [10] and König et al. [1] can be reconciled very well with the assumption that dried blood was again liquefied and/or blood clots were mobilised postmortem by an ablution and this newly mobilised blood was subsequently transferred to the Shroud. When examining the blood stains attributed to injuries from the *crown of thorns*, the most similar blood stain pattern to that on the Shroud was produced by the addition of oil to the hair [1].

To try and standardise our experiments, a certain sequence of arm movements had to be predetermined. We tried to involve many different movement axes within a time frame of 2 min; after this period of time, under the test conditions, the blood flow rate was significantly reduced due to the onset of coagulation. Of course, many other movement sequences are imaginable.

Obviously, the chosen experimental setup may be far from the actual course of events before and after the crucifixion of the TSM and his body being put in the Shroud. Nevertheless, our experiments revealed some interesting findings that may contribute new aspects to the discussion regarding the formation of blood stains on the TS.

Whilst the blood stain pattern on the left wrist of the TSM could be easily provoked by slowly moving the wrist and forearm upwards and to the ulnar side, it was really difficult to provoke a pattern of multiple branches of blood flows as seen on the TSM’s forearms. In all our experiments, the most effective way to provoke multiple bifurcations of blood flows was jerky and quick arm movements. More body hair seemed to facilitate the formation of a more branched pattern, but only slightly. The application of oily substances to the skin before applying blood significantly changed the way the blood flowed on the skin and facilitated the formation of branched streaks, obviously by way of reducing the blood’s surface tension. These observations were independent of anatomical characteristics of the probands and could be reproduced repeatedly.

What can we conclude from these experimental results regarding the hypotheses in the literature?

**The ‘bleeding at the cross’ hypothesis:** Blood stains on the forearms are the result of a vital bleeding from the *nailing wounds* whilst crucified [5, 7–9].

According to the results of our experiments, an elevated position of the arm at the time of blood flow—e.g. whilst hanging on the cross—may explain blood flows from the hands or wrists over the forearms to the elbows. However, it does not explain the formation of a multi-branched type of pattern. Our results also contradict the hypothesis of an “alteration of sagging and lifting” [7] of the body on the cross as causation for the branching of bloody streaks, as suggested by Bucklin [8] and Barbet [7], since branchings or bifurcations could only be provoked by jerky arm movements, which are hardly conceivable for a crucified man. Even if they somehow were possible, they most likely would have been avoided at any cost to minimise pain. Furthermore, the blood on the TSM would have mostly dried by the time of his removal from the cross (according to Bevilacqua et al. [18], this occurred 2 h after death and in a hot and dry environment); under these conditions, the formation of such well-defined blood stains, as observed on the TS, can not be assumed [1].

**The ‘postmortem bleeding’ hypothesis:** The blood stains on the forearms are the result of postmortem blood flows after unnailing und due to movements of the body during the preparation of the burial [6, 10].

In view of our results, this hypothesis seems more likely than the ‘bleeding at the cross’ hypothesis, since some of the influence factors we identified as favourable to a branching of blood flows may have played a role in the time between unnailing and burial. It is not known how the TSM was transported to the tomb, under what conditions and where he was possibly washed (in an atypical way?) and if the body was moved after washing and oiling. However, it seems plausible that his arms were exposed to passive jerky movements postmortem, and that the adding of water and oils may have also played a role (e.g. due to transporting the washed and oiled body from the place of burial preparation to the tomb), without this being able to be reconstructed exactly. Even if a postmortem formation of a branched blood stain pattern seems possible under such special circumstances, the multiple branchings as well as the clearly demarcated and well-defined shapes of the blood stains on the TS are striking and difficult to explain from a forensic pathologist’s point of view.

**The ‘scourge wounds’ hypothesis:** The presence of *scourge wounds* could have affected the blood stain pattern on the forearms [15].

According to the Gospels, Jesus was scourged (e.g. John 19:1, Mark 15:15 [3]). More than 100 stains in different locations on the TSM’s body have been attributed to scourging with different kinds of torture instruments [8, 15].

Faccini [15] proposed that the blood flows (from the *nailing wounds *on the hands) might have been influenced by *scourge wounds *on the forearms, without further explaining this line of reasoning. The scourging injuries could have resulted in a non-uniform swelling of soft tissue on the forearm, which could have drastically changed the relief of the skin’s surface and thus the blood flow pattern from the *nailing wounds*. However, such non-uniform swelling would have also resulted in an uneven contact area between the skin and the TS, giving evermore food for thought to the as yet unexplained manner of transfer of well-defined, seemingly undisturbed blood stains to the TS.

**Fig. 5:**
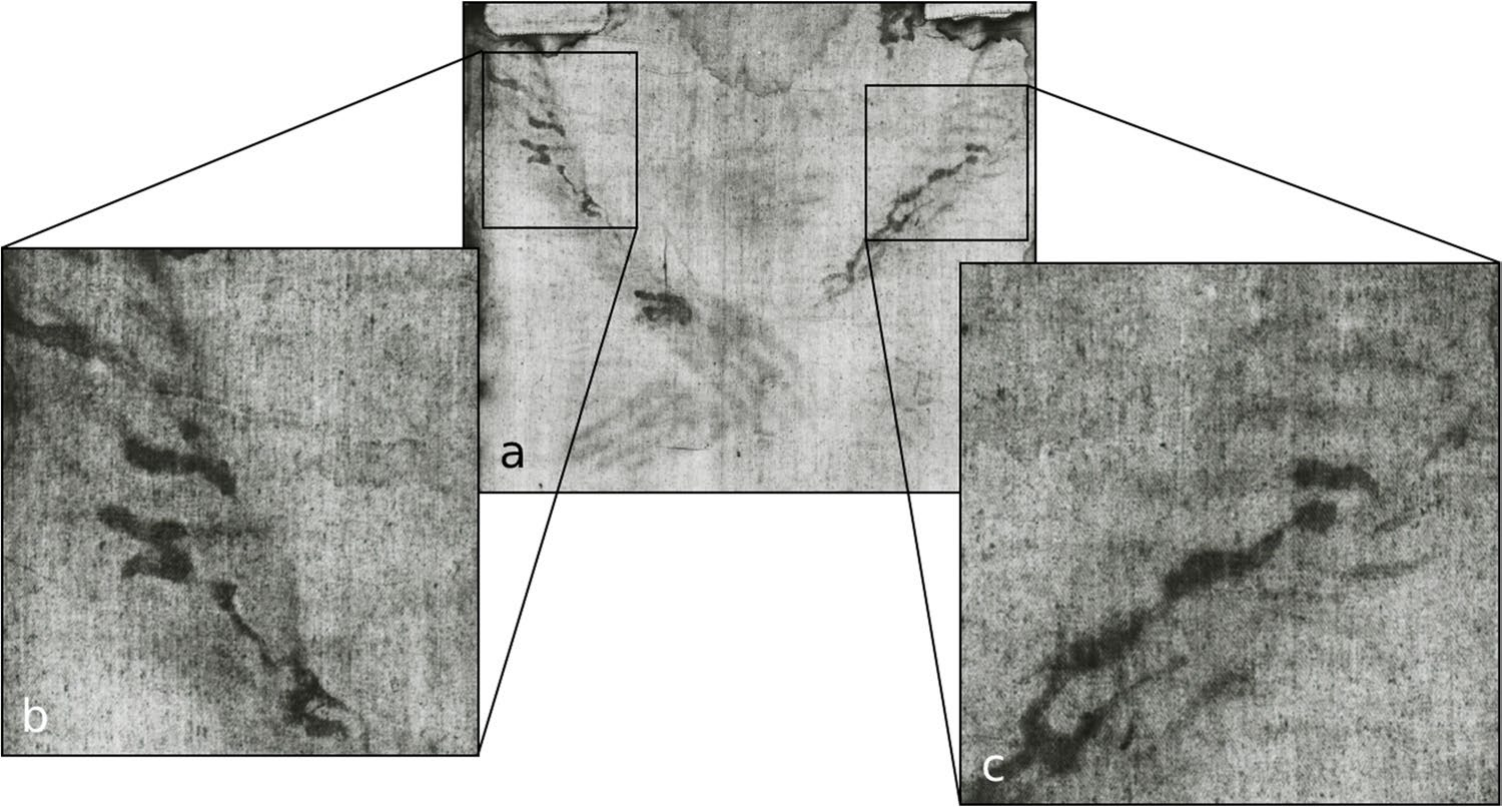
Scourge wounds and blood stains on the TSM’s forearms. **a** Forearms and hands on the TS, **b**, **c** closer look at the proximal forearms (photos from http://www.shroudphotos.com, ©Vernon Miller, 1978; cropped by the authors)

Whilst Faccini [15] kicked off the debate about the role of *scourge wounds* in modifying the directions of the blood flows on the forearms, she (like most other authors) assumed that the blood originated from the *nailing wounds* on the hands. However, the *scourging wounds* themselves could have been the source of the blood flows on the forearms. Scourging may not only cause superficial excoriations but also deeper, bleeding wounds. Looking closely at the TSM’s forearms, one can make out striped discolorations (attributed to *scourge wounds*, e.g. by Faccini [15]), running parallel to and being partly overlain by blood streaks (see Fig. 5).

It seems likely that the body of the wounded man was covered in blood during his way to Golgotha and even more so after his crucifixion. Active bleeding from all wounds would have ceased by the time of death at the latest, and all blood on the wounds or surrounding skin should have mostly dried by the time of burial. Under such conditions, a transfer of sharp edged, well-defined and continuous blood stains to the TS would not have been possible [1]. However, if the TSM was washed shortly before the body was wrapped in the Shroud, dried blood could have been liquidised and subsequently transferred to the TS [1, 10].

According to our previous experimental results [1], the transfer of well-defined blood stains after washing would be even more likely if oily substances were also used in a ritual preparation of the body before burial; under such simulated conditions, clearly demarcated blood stains could be produced [1]. Therefore, it is worth thinking about the possibility that the blood stains on the forearms do not (only) result from blood flowing from the *nailing wounds* but (also) originated from the *scourging wounds* on the forearms. In this context, it should be noted that in our experiments the proximal parts of the dorsal forearms showed (almost) no bloody rivulets after application of blood to the dorsal and palmar wrist area and various subsequent arm movements. Bleeding *scourge wounds* in this area of the forearm could, in our opinion, explain these blood stains better than blood originating from the hands or wrists alone (see Fig. 5).

## Conclusions

A blood flow pattern similar to the one depicted on the left wrist of the TSM could be easily simulated by applying blood to the dorsal side of the wrist and slowly turning the wrist to the ulnar side. However, this pattern is somewhat non-specific and may be attributed to the *nailing wound* on the hand; a differentiation between a formation antemortem (by the process of nailing, erection of the cross and the body hanging on the cross) and postmortem (unnailing and moving the body to the tomb) is not possible.

The simulation of a blood flow pattern with multiple branches similar to the blood stains on the TSM’s forearms required jerky arm movements and could be facilitated by oiled skin. These findings support the ‘postmortem bleeding’ hypothesis, since the relevant influence factors identified (jerky movements, oiled skin) may have played a role in the time between unnailing, possibly washing and oiling of the body and the burial.

However, in light of our findings, a further hypothesis should be discussed: If the body of the TSM was washed and oiled before burial, blood from deep scourging wounds on the forearms may have been newly liquidised and transferred to the TS. Therefore, the formation of the blood stain pattern on the forearms on the TS may not (only) have originated from the *nailing wounds*, but (also) from deep scourging wounds.

We have already pointed out the considerable limitations that all interpretations of the blood stain pattern on the TS entail [1]. At the moment, any interpretation can only be based on photographs of the TS, and there is little scientifically verified information concerning relevant details about the course of events before and after the crucifixion and what exactly happened with and to the TS around 2000 years ago and ever since. It is obvious that under these conditions the controversy of the origin of the blood stains on the TS can never be resolved (by scientific approaches). Nevertheless, our findings may contribute to the ongoing discussion on this interesting topic.

## Data Availability

Data sharing not applicable to this article as no datasets were generated or analysed during the current study.
